# Supplementary material for: Deletion of wfs1 Impairs Oligodendrocyte Precursor Cells Dorsal Distribution and Myelination Through the wfs1-hmgcs1 Axis in Zebrafish
Source: Biology (Basel). 2026 Aug 16;15(16):1402. doi: 10.3390/biology15161402 (PMC13510151; doi:10.3390/biology15161402)
Supplement: Supplementary file 1 [file biology-15-01402-s001.zip › File S1-Transcriptome sequencing data and differentially expressed genes (DEGs) in wfs1-- zebrafish at 5 dpf/1.GO/wfs1KOvsWT/DOWN/wfs1KOvsWT.down_GObar_bp.pdf]

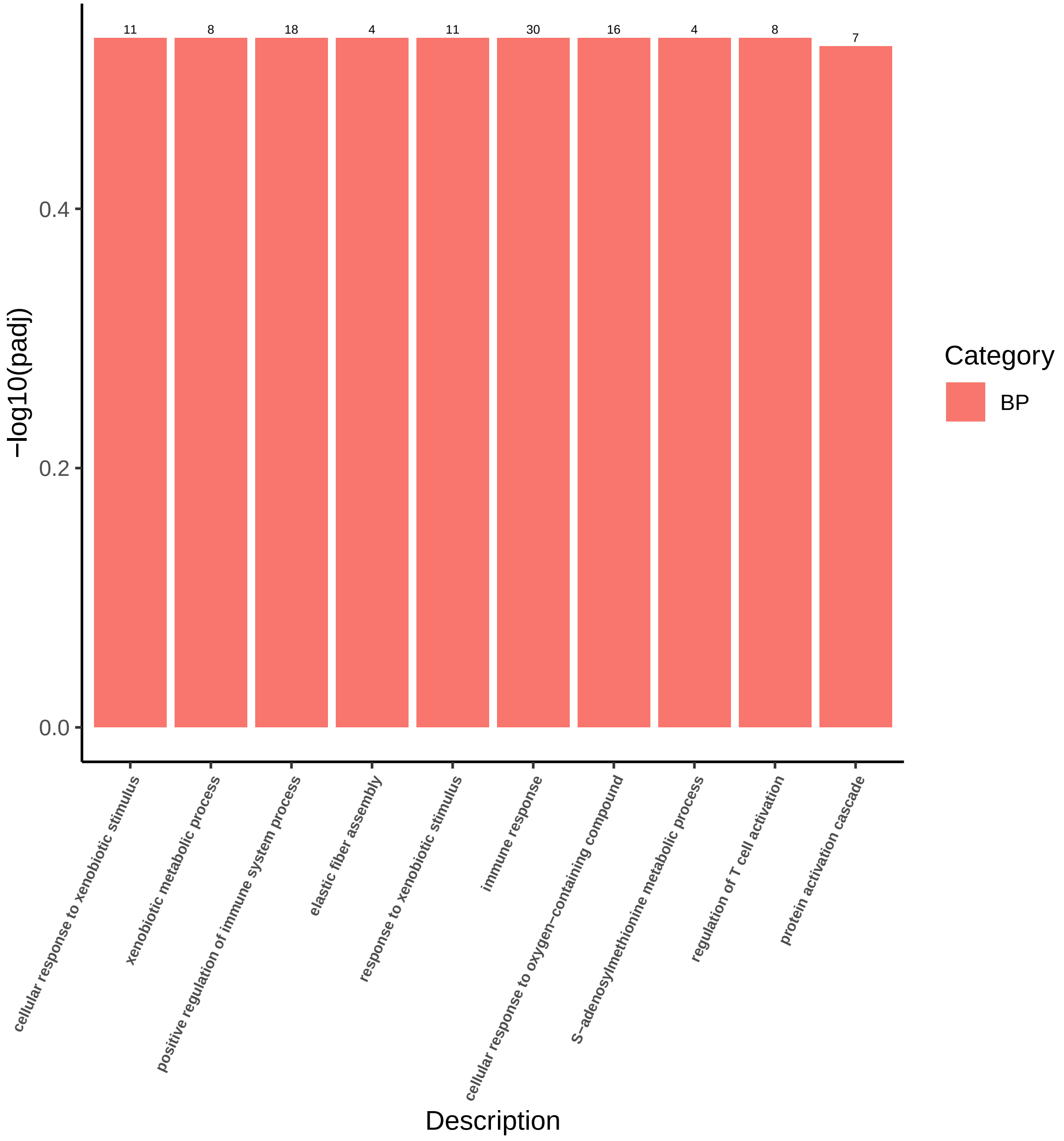

11
8
18
4
11
30
16
4
8
7
0.4
0.2
0.0
−log10(padj)
Category
BP
cellular response to xenobiotic stimulus
xenobiotic metabolic process
positive regulation of immune system process
elastic fiber assembly
response to xenobiotic stimulus
immune response
cellular response to oxygen−containing compound
S−adenosylmethionine metabolic process
regulation of T cell activation
protein activation cascade
Description
